# 

**Published:** 1833-01-01

**Authors:** 


### MEDICO-CHIRURGICAL REVIEW,

AND

### JOURNAL

OF

### PRACTICAL MEDICINE.

*(NEW SERIES.)*

VOLUME EIGHTEEN,

[1st of OCTOBER, 1832, to 30th of MARCH,]

1833.

VOL. XXII. of ANALYTICAL SERIES.

Nothing to index

EDITED BY JAMES JOHNSON, M.D.

PHYSICIAN EXTRAORDINARY TO THE KING,

*&c. &c. &c.*

Surgeon General's Office LIBRARY 57457

LONDON:

PUBLISHED BY S. HIGHLEY, 32, FLEET STREET,

AND WEBB STREET, ST. THOMAS'S HOSPITAL.

*Re-printed in New York, by Mr. Wood.*

1833.

WI
M675T

PRINTED BY G. HAYDEN,
Little College Street, Westminster.

### BIBLIOGRAPHICAL RECORD;

OR,

*Works received for Review since the last Quarter.*

1. A Treatise on the Epidemic Cholera, as it has prevailed in India; together with the Reports of the Medical Officers, made to the Boards of Bengal, Madras, and Bombay, &c. with a critical Examination of all the Works which have hitherto appeared on the Subject. By FRED. CORBYN, Esq. Surgeon on the Bengal Establishment. Octavo, pp. 389. Calcutta, 1832. Parbury and Co. Leadenhall Street, London.

---

2. Animal Mechanics, applied to the Prevention and Cure of Spinal Curvature and other Deformities. By T. SHELDRAKE. Part I. dedicated by permission to the King. One Vol. 8vo, pp. 347. September, 1832.

---

3. The Anatomy of the Horse; embracing the Structure of the Foot. By WILLIAM PERCIVALL, M.R.C.S. Veterinary Surgeon in the First Life Guards. One Vol. 8vo, pp. 454, price 20s. Longman and Co. 1832.

☞ *A highly valuable work for the veterinary student.*

---

4. The Law of Fire and Life Insurance and Annuities, with practical Observations, &c. By CHARLES ELLIS, of Lincoln's Inn, Esq. Barrister at Law. Octavo, pp. 263. 1832.

☞ *A very useful book for those whom it may concern.*

---

5. New Theory of the Influence of Variety in Diet in Health and Disease, accounting for many obscure Affections hitherto but little understood, and tending to elucidate the Nature and requisite Treatment of the existing Epidemic Cholera. By CHARLES CAMERON, Surgeon, R.N. Octavo, pp. 104, 1832.

---

6. A practical Treatise on Cholera, as it has appeared in various Parts of the Metropolis. By ALEXANDER TWEEDIE, M.R.C.S. and CHAS. GASELEE, M.R.C.S. &c. Octavo, pp. 79. October, 1832.

---

7. The Nature and Treatment of the Epidemic Cholera. By ROBERT VENABLES, A.M. &c. Octavo, pp. 52. Oct. 1832.

8. Four Lectures on the Study and Practice of Medicine; delivered, on different Occasions, in the University of London. By JOHN CONOLLY, M.D. late Professor, &c. in the University. Small 8vo, pp. 177. Oct. 1832.

☞ *We warmly advise the perusal of these four Lectures to every student of medicine. They will repay him tenfold, in moral as well as physical profit.*

---

9. A Dictionary of Practical Medicine; comprising General Pathology—the Nature and Treatment of Diseases, Morbid Structures, and the Disorders especially incidental to Climates, to the Sex, and to the different Epochs of Life; with numerous Prescriptions for the Medicines recommended—a Classification of Diseases according to Pathological Principles—a copious Bibliography, with References, and an Appendix of approved Formulæ; the whole forming a Library of Pathology and Practical Medicine, and a Digest of Medical Literature. By JAS. COPLAND, M.D. Consulting Physician to Queen Charlotte's Lying-in Hospital, Senior Physician to the Royal Infirmary for Diseases of Children, &c. Royal 8vo, pp. 336, and Appendix, p. 16, of Formulæ. Part I. Oct. 1832, price 9s.

☞ *The beginning of a most valuable work. See Review.*

---

10. A short Treatise on Cupping. By MONSON HILLS, Cupper to Guy's Hospital. Duodecimo, pp. 88. Cox, Borough, price 3s. 6d., 1832, with three Lithographic Plates.

☞ *This little volume contains many ingenious suggestions and original observations.*

---

11. Observations on the Powers and Effects of Cold, as a Cause of Disease; with some Remarks on the best Means of preventing its Morbid Effects. By JOHN CLENDINNING, M.D. of Edinburgh and Oxford, &c. Octavo, pp. 39. Reprinted from the Med. and Phys. Journal.

☞ *A very practical and ingenious essay on an interesting subject. See Periscope.*

12. Elements of Materia Medica and Therapeutics, including the recent Discoveries and Analyses of Medicines. By A. T. THOMSON, M.D. &c. Professor of Materia Medica in the University of London, &c. Octavo, pp. 747. Vol. I. Longman's 1832.

☞ *This valuable work in our next.*

---

13. A Critical Enquiry respecting a new Membrane in the Eye, discovered by Mr. George Hunsley Fielding, and described by him in a Lecture delivered at Oxford, &c. By WILLIAM GORDON, F.L.S. &c. Octavo, pp. 42, sewed. Oct. 1832.

☞ *This is a sharp examination, and, as is averred, a refutation of Mr. Fielding's discovery.*

---

14. The Substance of the Official Medical Reports upon the Epidemic called Cholera, which prevailed at Dantzick, &c. By JOHN HAMETT, M.D. Octavo, pp. 190. Nov. 1832.

---

15. Essay on the Natural History, Origin, Composition, and Medical Effects of Mineral and Thermal Springs. By MEREDITH GAIRDNER, M.D. Octavo, pp. 420. Edinburgh and London, 1832.

☞ *This is a work of extreme research, ingenuity and utility.*

---

16. A Lecture, introductory to the Course of Anatomy and Physiology, delivered in the Medical School, Aldersgate Street, London, upon the opening of the Session, 1832. By ROBERT TODD, M.A. Lecturer on Anatomy, pp. 20. Nov. 1832.

☞ *An appropriate and sensible address.*

---

17. An Essay on the Structure and Functions of the Skin; with Observations on the Agency of Atmospheric Vicissitudes, through the Medium of the Skin, in the Production of Affections of the Lungs, Liver, Stomach, Bowels, &c. By WM. WOOD, M.D. Physician to the Newport Dispensary. Octavo, pp. 172. Nov. 1832.

---

18. An Account of the first Meeting of the Provincial Medical and Surgical Association, held in the Board-Room of the Worcester Infirmary, July the 19th, 1832; containing an Address delivered by CHARLES HASTINGS, M.D. together with a correct Report of the Proceedings of the Meeting, pp. 46. Nov. 1832.

☞ *Already 267 members have joined the Association, and success appears certain.*

---

19. The Cyclopædia of Practical Medicine, for September, October and November, viz. Nos. IX., X., and XI.

☞ *This national work has now got as far in the alphabet as* IND, *and keeps up its reputation. The pressure of temporary matters has prevented our devoting as much attention to particular subjects as we intended; but the great circulation of the work renders this of less consequence.*

---

20. Description of the Appearances observed in a Case of double Uterus, in which Impregnation had taken place, with Remarks, &c. By ROBERT LEE, M.D. Octavo, pp. 36, with Plates. Nov. 1832.

---

21. Practical Remarks on the Epidemic Cholera, which at present prevails in Belfast and Vicinity. By THOMAS THOMPSON, M.D. Surgeon R.N. Octavo, pp. 35. Nov. 1832.

---

22 Tables for the Chemical Analysis of Incrganic Bodies. Quarto, pp. 15. Maclachlan and Stewart, Edinburgh, Nov. 1832, price 2s. 6d.

☞ *Collected with great care, and compressed with great art.*

---

23. A Lecture on the Nature, Causes, and Prevention of Cholera, delivered at the Ivanhoe Baths' Assembly Rooms, *Ashby-de-la-Zouche*, on Monday, 17th Sept. 1832, in Aid of the Funds of the Cholera Provident Society, and published at the particular request of the Board of Health of that Place. By JAMES M. S. KENNEDY, M.D. Royal Octavo, pp. 36. 1832.

*See Periscope.*

---

24. An Address delivered in King's College, London, at the Commencement of the Medical Session, October 1st, 1832. By J. H. GREEN, F.R S. &c. Octavo, pp. 43. Nov. 1832.

☞ *A most learned and eloquent address, as might be expected.*

---

25. A Treatise on Inflammations; explaining their Pathology, Causes, Consequences, and Treatment, with their Effects on the various Textures of the Body, being an Extension of a Dissertation on "Inflammation of the Membranes," to which the Jacksonian Prize, for the Year 1828, was awarded by the Royal College of Surgeons. By Geo. Rogerson, Surg. of Liverpool. Vol. I. pp. 459. Octavo, London, Nov. 1832.

☞ *The second volume will shortly be published.*

---

26. The Heliotrope, or Pilgrim in Pursuit of Health. Canto the first. Octavo, pp. 57. London, Dec. 1832.

☞ *The author appears to have been an invalid who voyaged to Genoa, and thence travelled to Pisa, for his health. The poetry is in imitation of Childe Harold, and is highly descriptive of the scenes through which the pilgrim passed.*

---

27. Floriculture, comprising the general Management and Propagation of Stove, Green-house, and hardy Herbaceous Plants, hardy Trees, and Shrubs. By Mr. Josh. Mantell, Surgeon. Royal 8vo, pp. 36, 1832.

☞ *By the simple arrangement here adopted, Mr. M. has been able to comprise in a few pages the substance of Sweet's Botanical Cultivator, an 8vo volume, of* 700 *pages.*

---

28. On the Extermination or Annihilation of Cholera. A Letter to Lord Melbourne from Dr. Sanders, of Edinb.

---

29. Plain Rules for the Treatment of Spasmodic Cholera on Board of Ships. Intended for the Use of Commanders of Vessels at Sea, when Medical Aid cannot be procured. By Edward Greenhow. Pp. 11, price 3d.

☞ *Very well adapted to the end designed.*

---

30. Cases of Cholera collected at Paris, in the Month of April, 1832, in the Wards of Messrs. Andral and Louis, at the Hôpital de la Pitiè. By James Jackson, jun. Boston, 1st Nov. 1832.

☞ *These cases (*60 *in number) have been scrupulously recorded at the bedside, with dissections, therapeutic means, &c. They form a valuable record, where fidelity and minute accuracy are so much required.*

31. A System of Materia Medica and Pharmacy, including Translations of the Edinburgh, London, and Dublin Pharmacopœias. By John Murray, M.D. &c. Lecturer on Chemistry, Materia Medica, and Pharmacy. *Sixth Edition*, adapted to the present State of Chemical and Medical Sciences. By Jno. Murray, M.D. Fellow of the Royal College of Surgeons, &c. Octavo, pp. 886, 1832, price 20s.

☞ *Some notice of this valuable work in our next Number.*

---

32. The Cyclopædia of Practical Medicine, Part XII. Dec. 1832.

☞ *The work has now reached as far as* "Inflammation," *and we perceive in the last two parts some highly valuable articles.*

---

33. Elements of Surgery. By Robert Liston, &c. Part the Third. Octavo, pp. 409, Dec. 1832.

*In our next.*

---

34. A Treatise on the Urethra; its Diseases, especially Stricture, and their Cure. By Benjamin Phillips. Octavo, pp. 317, with a Plate, 8s. bds.

---

35. Elements of Diagnosis, general Pathology, and Therapeutics. By Robert Norton, M.D. M.R.C.S. Octavo, pp. 100, with Appendix. Jackson, Borough.

☞ *Most of the materials of this little volume are derived from memoranda and recollections of unpublished Lectures of the late Dr. Armstrong. The author appears to be exceedingly religious, and has appended to his book some account of the last days and sentiments of Dr. Bateman.*

---

36. Medical Botany; or Illustrations and Descriptions of the Medicinal Plants of the London, Edinburgh, and Dublin Pharmacopœia, including Poisonous Vegetables, &c. By Dr. Stephenson and Mr. Churchill. New Edition, edited by Professor Gilbert Burnett. Nos. I., II., and III. John Churchill, Princes Street, Soho, Oct. Nov. and Dec. 1832, price 2s. 6d. each Number.

☞ *This new edition, greatly improved in value and reduced in price, will be found a most acceptable present to the botanical student, and the lover of botany in general.*

